# Complete chloroplast genome sequence of *Stephania kwangsiensis* (Menispermaceae), a rare and critically endangered species endemic to China

**DOI:** 10.1080/23802359.2019.1698984

**Published:** 2019-12-12

**Authors:** Yancai Shi, Bingbing Liu

**Affiliations:** a Institute of Loess Plateau, Shanxi University, Taiyuan, China;; b Guangxi Institute of Botany, Guangxi Zhuang Autonomous Region and Chinese Academy of Sciences, Guilin, China

**Keywords:** *Stephania*, chloroplast genome, phylogenetic analysis

## Abstract

*Stephania kwangsiensis* (Menispermaceae) is a rare and critically endangered species endemic to China. Here, we first report and characterize its complete chloroplast genome sequence based on Illumina paired-end sequencing data. The complete plastid genome was 156,624 bp, which contained inverted repeats (IR) of 24,348 bp separated by a large single-copy (LSC) and a small single copy (SSC) of 87,759 bp and 20,169 bp, respectively. The cpDNA contains 132 genes, comprising 85 protein-coding genes, 37 tRNA genes, eight rRNA genes, and two processed pseudogenes. The overall GC content of the plastome is 38.4%. The phylogenetic analysis of 18 selected chloroplast genomes demonstrated that *S. kwangsiensis* is related to the congeneric *S. japonica*.

*Stephania kwangsiensis* H. S. Lo., a perennial deciduous herbaceous liana which belongs to the family Menispermaceae, is a rare and critically endangered species endemic to China. It is limited distributed throughout the Guangxi Zhuang Autonomous Region and Yunnan Province of China (Min and Zhong 1980). However, because of the limited population and narrow distribution area, *S. kwangsiensis* is treated as rare and endangered species in China and has been registered on the China Species Red List (Fu 1991). It is thus urgent to take effective measures to conserve this critically endangered and precious species. Herein, we first report and characterize the complete plastome of *S. kwangsiensis* based on Illumina paired-end sequencing data, which will contribute to the further studies on its genetic research and resource utilization. The annotated cp genome of *S. kwangsiensis* has been deposited into GenBank with the accession number MN654112.

In this study, *S. kwangsiensis* was sampled from in Guangxi Zhuang Autonomous Region of China, located at 110°21′34″ E, 22°47′11″ N. A voucher specimen (Y.-C. Shi et al. H1128) was deposited in the Guangxi Key Laboratory of Plant Conservation and Restoration Ecology in Karst Terrain, Guangxi Institute of Botany, Guangxi Zhuang Autonomous Region and Chinese Academy of Sciences, Guilin, China. The experiment procedure is as reported in Zhang et al. (2019). Around 2 Gb clean data were used for the cp genome de novo assembly by the program NOVOPlasty (Dierckxsens et al. 2017) and direct-viewing in Geneious R11 (Biomatters Ltd., Auckland, New Zealand). Annotation was performed with the program Plann (Huang and Cronk 2015) and Sequin (http://www.ncbi.nlm.nih.gov/).

The chloroplast genome of *S. kwangsiensis* is a typical quadripartite structure with a length of 156,624 bp, which contained inverted repeats (IR) of 24,348 bp separated by a large single-copy (LSC) and a small single copy (SSC) of 87,759 bp and 20,169 bp, respectively. The cpDNA contains 132 genes, comprising 85 protein-coding genes, 37 tRNA genes, eight rRNA genes, and two processed pseudogenes. Among the annotated genes, 15 of them contain one intron (atpF, ndhA, ndhB, rps16, rpoC1, petB, petD, rpl16, rpl2, trnA-UGC, trnI-GAU, trnG-GCC, trnK-UUU, trnL-UAA, and trnV-UAC), and three genes (clpP, rps12, and ycf3) contain two introns. The overall GC content of the plastome is 38.4%.

To identify the phylogenetic position of *S. kwangsiensis*, phylogenetic analysis was conducted. A neighbor-joining (NJ) tree with 1000 bootstrap replicates was inferred using MEGA version 7 (Kumar et al. 2016) from alignments created using the MAFFT (Katoh and Standley 2013) using plastid genomes of 17 species. It showed the position of *S. kwangsiensis* is related to the congeneric *S. japonica* (Figure 1). Our findings can be further used for population genomic and phylogenomic studies of Menispermaceae. It will also provide fundamental data for the conservation, utilization, and management of this rare species.

**Figure 1.**
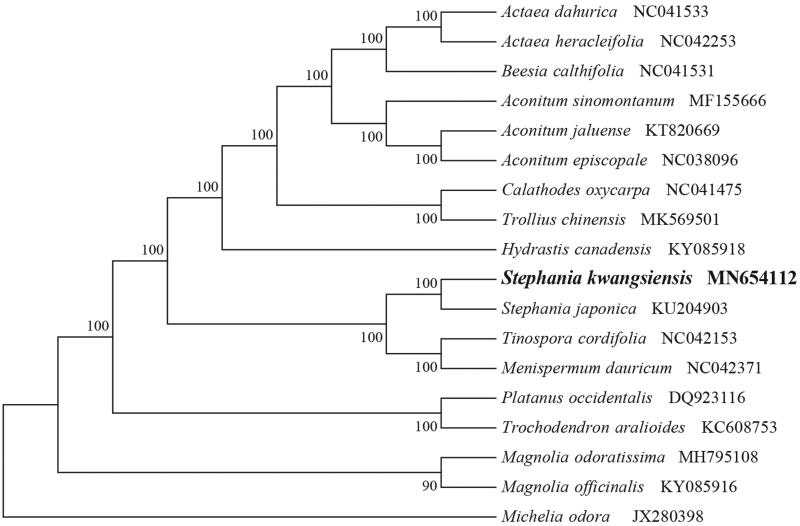
NJ phylogenetic tree of *S. kwangsiensis* with 17 species was constructed by chloroplast plastome sequences. Numbers on the nodes are bootstrap values from 1000 replicates. *Michelia odora* was selected as outgroups.
